# Unilateral renal atrophy 4 years after renal sympathetic denervation: a case report

**DOI:** 10.1097/HJH.0000000000003350

**Published:** 2023-01-03

**Authors:** Victor J.M. Zeijen, Alexander Hirsch, Michiel G.H. Betjes, Joost Daemen

**Affiliations:** a Department of Cardiology; b Department of Radiology and Nuclear Medicine; c Department of Internal Medicine, Section Nephrology and Transplantation, Erasmus University Medical Center, Rotterdam, the Netherlands

**Keywords:** ambulatory, antihypertensive agents, atrophy, blood pressure, blood pressure monitoring, glomerular filtration rate, hypertension, magnetic resonance angiography, renal artery obstruction, sympathectomy, ultrasonography

## Abstract

Renal sympathetic denervation (RDN) carries a low risk of renal artery stenosis, and most cases occur within the first year. However, limited data are available on long-term incidence. Here, we present a case of a 68-year-old woman who underwent radiofrequency RDN for resistant hypertension. Ambulatory blood pressure improved following RDN and uptitration of antihypertensive drugs. Between year 3 and 4 after RDN, eGFR reduced from 87 to 50 ml/min per 1.73 m^2^. Ultrasound imaging revealed left renal atrophy, while subsequent magnetic resonance angiography showed a haemodynamically significant stenosis of the left renal artery. The patient remained in good clinical condition with stable blood pressure, while eGFR mildly deteriorated during a 6-year follow-up period. This case of renal artery stenosis occurred in a patient with multiple risk factors. A causal relationship to the RDN procedure cannot be confirmed nor ruled out. Long-term surveillance for adverse events should be considered in all RDN patients.

## INTRODUCTION

Renal sympathetic denervation (RDN) demonstrated to significantly lower ambulatory SBP by 4–8 mmHg [1–6]. Low rates of RDN-related adverse events were reported on the short as well as the long-term [1–8]. Accordingly, renal function (as measured by estimated glomerular filtration rate; eGFR) remained stable on the short-term post RDN [1–6]. On the long term, only a moderate drop in eGFR was observed, most likely reflecting the natural course of renal function over time in patients with a history of (severe) hypertension [7,8].

Magnetic resonance angiography (MRA) emerged as a feasible and reliable technique for the assessment of renal artery dimensions (as compared to intravascular ultrasound) in patients undergoing RDN [9]. Routine MRA measurements showed no decline in renal artery lumen area and diameter and no haemodynamically significant renal artery stenosis (RAS) occurred throughout 1 year post RDN (*n* = 27 patients) [10]. A recent meta-analysis (*n* = 5769 patients) demonstrated that renal artery damage occurred in only 0.45% of the patients (0.33% stenosis, 0.12% dissections) within a median follow-up period of 6 months following RDN (range: 1–36 months) [11]. In patients with renal artery damage, 92% underwent repeat renal artery intervention, resulting in an annual incidence rate for renal artery reintervention of 0.20% [11]. Whereas most cases were discovered early, 21% were detected later than 1 year following RDN (range: 14–33 months) [11].

As of to date, there is a lack of data on the incidence of late RAS in patients who underwent RDN. Here, we present a case of renal atrophy due to RAS occurring 4 years post RDN. The aim of this case report is to raise awareness for any clinical signs of late RAS in patients who previously underwent RDN.

## CASE REPORT

**FIGURE 1:**
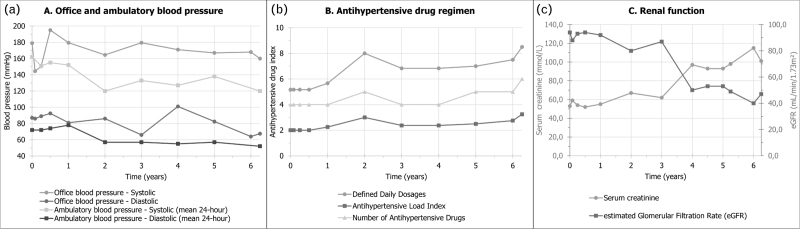
(a) Blood pressure, (b) prescribed antihypertensive drug regimen and (c) renal function over time.

A 68-year-old woman with a history of hypercholesterolemia, type 2 diabetes mellitus, former-smoking and resistant hypertension was screened for RDN. At baseline, mean office BP was 179/87 mmHg and mean 24-h ambulatory BP was 162/72 mmHg (Fig. 1a), while the patient was prescribed metoprolol 100 mg, valsartan 160 mg, amlodipine 10 mg and hydrochlorothiazide 12.5 mg, equivalent to a number of 5.2 defined daily doses (DDD; Fig. 1b). Renal function (eGFR) was 94 ml/min per 1.73 m^2^ (Fig. 1c) and MRA revealed no RAS or other abnormalities of the renal arteries (Table 1). Treatable secondary causes for hypertension were ruled out based on laboratory sampling and MRI.

**TABLE 1:** Renal artery magnetic resonance angiography parameters

	Baseline	6 months	1 year	4 years
Left renal artery				
Mean lumen area (mm^2^)	13	9	12	10
Lumen area stenosis^a^ (%)	23	31	30	89
Mean effective diameter (mm)	4	3	4	4
Effective diameter stenosis^b^ (%)	12	16	15	66
Maximal diameter (mm)	5	4	5	5
Minimal diameter (mm)	3	2	3	1
Right renal artery				
Mean lumen area (mm^2^)	12	9	10	13
Lumen area stenosis^a^ (%)	26	28	17	29
Mean effective diameter (mm)	4	3	4	4
Effective diameter stenosis^b^ (%)	13	14	7	16
Maximal diameter (mm)	6	4	5	5
Minimal diameter (mm)	3	3	3	3

a Percentage stenosis was calculated using the following formula: 1 – (minimal lumen area / average lumen area).

b Percentage stenosis was calculated using the following formula: 1 – (minimal effective diameter / average effective diameter).

The patient underwent transfemoral RDN using the radiofrequency multielectrode Symplicity Spyral catheter (Medtronic, Minneapolis, Minnesota, USA) in context of the ASORAS study [12]. Four and six ablations were applied to the left and the right renal artery, respectively. The patient was discharged the next morning in good clinical condition.

**FIGURE 2:**
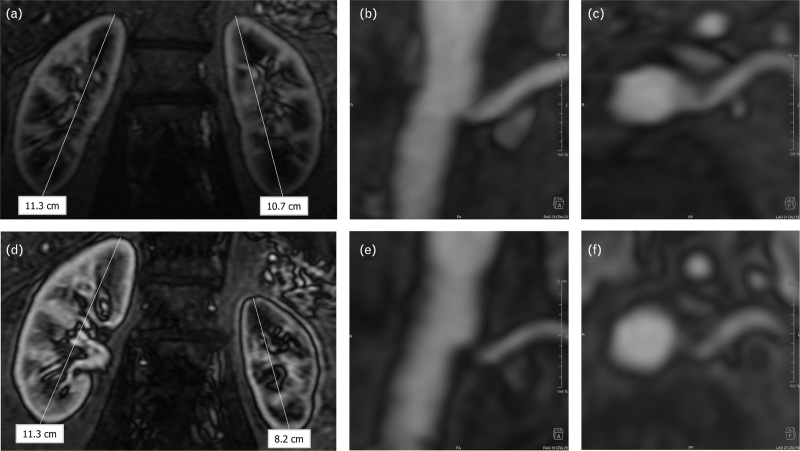
Magnetic resonance angiography of the kidneys and renal arteries. (a—c) Coronal view of the right and left kidney (a) and a coronal/transversal (b/c) view of the ostium of the left renal artery at 1 year following renal denervation. No parenchymal, cortical or vascular abnormalities of the kidneys were observed. (d—f) Coronal view of the right and left kidney (d) and a coronal/transversal (e/f) view of the ostium of the left renal artery at 4 years following renal denervation. Reductions in the diameter and cortical volume of the left kidney were observed (d), caused by a significant stenosis of the ostium of the left renal artery (e/f).

Protocolized follow-up was performed at 1, 3 and 6 months. At 6 months, on a stable antihypertensive drug regimen, mean 24 h ambulatory BP was 155/74 mmHg, reflecting a –7/+2 mmHg change as compared to baseline (Fig. 1a). Antihypertensive drug treatment was subsequently intensified and eGFR remained stable (Fig. 1b,c). Renal artery MRA at 6 and 12 months post RDN showed no signs of RAS (Fig. 2, Table 1).

Four years after the RDN procedure, the patient was diagnosed with a progressive decline in kidney function (eGFR of 50 ml/min per 1.73 m^2^, representing a change of –37 ml/min per 1.73 m^2^ as compared to the visit 1 year before, Fig. 1c). Ultrasound imaging revealed atrophy of the left kidney, while no significant changes to the right kidney were observed. Consequently, renal artery MRA was performed, which confirmed left renal atrophy due to a haemodynamically significant stenosis (89% lumen area stenosis) of the ostium of the left renal artery (Fig. 2, Table 1). On the basis of multidisciplinary consensus, no renal artery intervention was performed, as renal atrophy was already present at the time the stenosis was discovered. Renal function and BP were closely monitored, and the patient remained in a good clinical condition up until 6 years post RDN (Fig. 1).

## DISCUSSION

Our patient developed unilateral RAS 4 years after RDN, raising the possibility of a causal relationship to the RDN procedure. Following RDN, an annual incidence of renal artery damage of 0.20% was observed in a meta-analysis of single-arm studies [11]. However, no adequately powered randomized studies have been performed on the topic. Therefore, the additional risk of RDN for the development of RAS cannot be distinguished from the general risk of RAS. The general prevalence of renovascular disease (defined as haemodynamically significant stenosis or total occlusion as diagnosed by Doppler ultrasound) was reported as 6.8% in a population-based cohort study in elderly patients (age > 65 years; *n* = 870) [13]. Furthermore, higher age, higher office SBP and lower high-density lipoprotein cholesterol were identified as independent risk factors for renovascular disease [13]. On the basis of these literature findings and the risk profile of the present case, the risk of RAS would still have been substantial in this patient, even if she had not undergone RDN. Altogether, we cannot confirm nor rule out a causal relationship between the RDN procedure and the occurrence of RAS 4 years later.

The current case example emphasizes the importance of routine clinical follow-up of patients treated with new technologies such as RDN. Standardized BP measurement and routine renal function testing can thereby guide early identification of serious adverse events. Noninvasive renal artery imaging should be considered in case of an unexplained increase in BP or an unexplained decline in renal function. In the current case, laboratory testing indicated new-onset chronic kidney disease and consequentially triggered the performing of additional imaging. On the basis of these clinical findings, more strict BP control was attempted in this patient, together with more frequent renal function assessment. In general, the main aims of adequate follow-up of RDN patients and (suspected) adverse events include both adequate diagnostics and therapy in individual patients and the ability of reporting common as well as rare adverse events for scientific purposes. On the basis of the latter, it is recommended to include all patients treated with new technologies in registry-based studies with a standardized follow-up schedule.

In conclusion, renal artery damage following RDN is considered a rare complication, which is usually discovered within 1 year following the procedure. We present a case of a patient who developed unilateral RAS and consequent renal atrophy 4 years after the index procedure. It remains elusive whether this adverse event has a causal relationship with the RDN procedure. By any means, these findings emphasize the importance of vigilance for late complications in patients who previously underwent RDN.

## ACKNOWLEDGEMENTS

None.

### Conflicts of interest

V.Z. received institutional grant/research support from ReCor Medical. J.D. received institutional grant/research support from Astra Zeneca, Abbott Vascular, Boston Scientific, ACIST Medical, Medtronic, Microport, Pie Medical and ReCor medical. All other authors declare no competing interests.
